# Functional and clinical evidence for two novel heterozygous *BUB1B* variants and their value in precision genetic counseling for recurrent pregnancy loss

**DOI:** 10.3389/fendo.2026.1838559

**Published:** 2026-06-26

**Authors:** Tian-ying Wei, Ming-xian Kang, Ge-han Zhang, Jing Zhang, Jia-en Liu, Jing Ma, Ya-ping Tian, Hua-ying Hu

**Affiliations:** 1 Beijing Jiaen Hospital, Heen Life Medical Research Institute, Beijing, China; 2 Medical Innovation Research Division of Chinese People's Liberation Army (PLA) General Hospital, Beijing, China; 3 Prenatal Diagnosis Center, Shijiazhuang Obstetrics and Gynecology Hospital, Hebei Key Laboratory of Maternal and Fetal Medicine, Shijiazhuang, Hebei, China; 4 Hebei Key Laboratory of Reproductive Medicine, Hebei Reproductive Health Hospital, Shijiazhuang, Hebei, China

**Keywords:** aneuploidy, *BUB1B*, genetic diagnosis, premature chromatid separation, recurrent pregnancy loss

## Abstract

**Introduction:**

Recurrent pregnancy loss (RPL) is a common reproductive disorder with largely unexplained etiology. Premature chromatid separation (PCS), a cytogenetic manifestation of chromosomal instability, is linked to spindle assembly checkpoint (SAC) dysfunction. While biallelic *BUB1B* mutations cause mosaic variegated aneuploidy syndrome, the contribution of heterozygous variants to RPL remains uncertain.

**Methods:**

Here, we investigated two unrelated families with unexplained RPL. Chromosomal microarray analysis of six miscarriage specimens revealed random aneuploidies. Whole-exome sequencing identified two novel heterozygous *BUB1B* variants—c.2164T>C (p.W722R) and c.2215G>T (p.A739S)—in the probands, confirmed by Sanger sequencing and segregation analysis. Agarose gel electrophoresis of RT-PCR products was performed to assess *BUB1B* mRNA expression, and Western blot was used to evaluate BUBR1 protein levels, in peripheral blood lymphocytes of both probands. Twenty healthy controls (10 male, 10 female) without history of pregnancy loss were enrolled for cytogenetic comparison. Cytogenetic analysis showed significantly elevated PCS rates in probands’ lymphocytes compared to controls, as detected by G-banding and centromere-specific FISH.

**Results:**

Two novel heterozygous *BUB1B* missense variants (c.2164T>C [p.W722R] and c.2215G>T [p.A739S]) were identified in the two probands. Both variants were classified as variants of uncertain significance (VUS) per ACMG/AMP guidelines. Significantly elevated PCS rates were confirmed in peripheral blood lymphocytes of both probands by G-banding and centromere-specific PNA-FISH. Agarose gel electrophoresis and Western blot analyses revealed a trend toward reduced *BUB1B* mRNA and BUBR1 protein expression in both variant carriers, providing preliminary functional support for a haploinsufficiency mechanism.

**Discussion:**

These findings provide the first clinical evidence suggesting that heterozygous *BUB1B* variants may impair SAC fidelity, leading to somatic PCS and recurrent embryonic aneuploidy, thereby contributing to sporadic RPL. Our study highlights *BUB1B* as a candidate gene for genetic screening in idiopathic RPL and underscores the importance of SAC integrity in early embryogenesis.

## 1 Introduction

Chromosomal instability (CIN) is a major contributor to early human embryonic developmental failure and spontaneous miscarriage (1). Among the various CIN-associated syndromes, premature chromatid separation (PCS) represents a distinctive cytogenetic phenotype characterized by the untimely separation of sister chromatids during metaphase, with clearly discernible centromeric regions (2). PCS serves not only as a defining feature of rare genetic disorders such as premature chromatid separation/mosaic variegated aneuploidy (PCS/MVA) syndrome (MIM #257300 and #176430) (3, 4), but has also been increasingly linked to sporadic recurrent pregnancy loss (RPL) (5–7). Evidence indicates that dysfunction of the spindle assembly checkpoint (SAC)—a critical surveillance mechanism ensuring accurate chromosome segregation—is the primary driver of PCS and subsequent aneuploidy (8, 9). The SAC kinase BUBR1, encoded by the *BUB1B* gene, plays an essential role in this process.

It is well established that biallelic pathogenic variants in *BUB1B* cause PCS/MVA syndrome, a severe autosomal recessive disorder typically presenting with intrauterine growth restriction, microcephaly, and a high predisposition to childhood cancers (3, 10, 11). In contrast, the clinical consequences of monoallelic (heterozygous) *BUB1B* variants remain incompletely understood. Although heterozygous carriers do not develop full-blown MVA syndrome, several studies have reported that they often exhibit a detectable PCS phenotype in peripheral blood lymphocytes and may be at increased risk for reproductive challenges, including infertility and RPL (12, 13). Notably, recent work has even associated deleterious heterozygous *BUB1B* variants with premature ovarian insufficiency (POI), further broadening their implications for reproductive health (12). Nevertheless, direct evidence connecting specific heterozygous *BUB1B* variants to recurrent embryonic aneuploidy in RPL patients—particularly in Chinese populations—remains scarce. Moreover, although current international guidelines (e.g., ESHRE 2018) recommend genetic evaluation in RPL, *BUB1B* is not yet included in routine screening panels (5).

In this study, we performed comprehensive genetic analyses on two unrelated families affected by unexplained RPL to identify underlying pathogenic variants. We integrated multiple approaches—including next-generation sequencing (NGS), genome-wide microarray, conventional karyotyping, and centromere-specific fluorescence *in situ* hybridization (FISH)—to systematically assess genomic variants, the PCS phenotype in peripheral blood, and chromosomal constitution of the products of conception. We identified two novel heterozygous variants in *BUB1B* and, for the first time in the context of RPL, associated these variants with both a significantly elevated frequency of PCS in carrier lymphocytes and recurrent random aneuploidy in miscarried embryos. Our findings provide new insights into the genetic etiology of RPL, highlight the importance of *BUB1B* screening in RPL patients exhibiting PCS, and lay the groundwork for precision genetic counseling and tailored assisted reproductive interventions. Variant interpretation was conducted in strict accordance with the American College of Medical Genetics and Genomics (ACMG) guidelines (14), supported by multiple in silico prediction tools including SIFT (15), PolyPhen-2 (16), and MutationTaster (17).

## 2 Results

### 2.1 Family characteristics and chromosomal microarray analysis of products of conception

**Figure 1:**
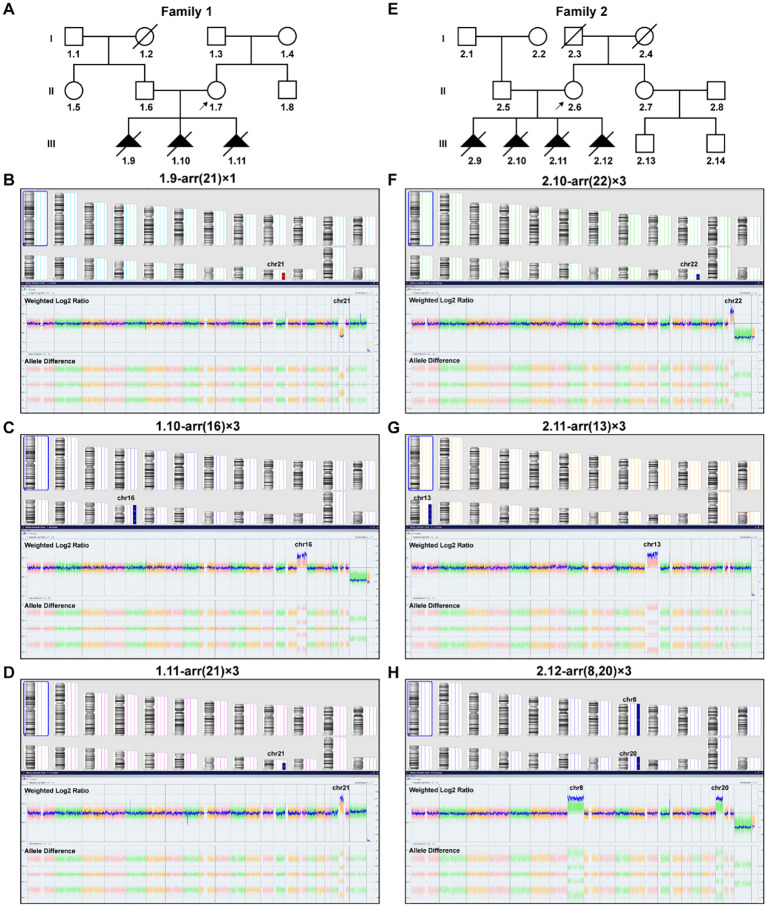
Pedigrees and chromosomal microarray results of products of conception from two RPL families. **(A, E)** Pedigrees of Family 1 and Family 2, respectively. Arrows indicate the probands. Black triangles denote individuals with documented embryonic arrest. **(B–D)** Genome-wide copy number profiles from chromosomal microarray analysis (CMA) of three miscarriage samples from the proband in Family 1. Each panel represents one sample. Blue segments indicate chromosomal gains (duplications), and red segments indicate losses (deletions). Each sample exhibits a distinct aneuploidy. **(F–H)** Genome-wide CMA profiles of three miscarriage samples from the proband in Family 2. Similarly, each sample shows a different aneuploid karyotype.

This study included two unrelated Han Chinese families with recurrent pregnancy loss (RPL) (Family 1 and Family 2). Each family comprised a female proband who had experienced three or more unexplained miscarriages, her spouse, and selected additional family members (**Figures 1A, E**). Both probands were confirmed to have no history of gestational infections or exposure to known teratogens. Their baseline clinical and reproductive histories are summarized in **Table 1**.

**Table 1:** Baseline clinical and reproductive characteristics of the probands.

Family	MC	Uterine/anatomical abnormalities	GP	Gestational weeks at arrest
Family 1	29 days	None	G3P0	6, 7, 10
Family 2	30 days	None	G4P0	7, 9, 6, 8

To investigate the potential genetic basis underlying embryonic arrest in these probands, we performed chromosomal microarray analysis (CMA) using the Affymetrix CytoScan™ 750K platform on six products of conception collected from the two probands. The results revealed distinct aneuploidies in all six samples (**Figures 1B–D**, **1F–H**), with a frequency markedly higher than that observed in the general population. The abnormalities included trisomies (+13, +16, +21, +22), monosomy (−21), and double trisomy (+8 and +20). No consistent pattern was observed across the samples, indicating random segregation errors (9), which strongly suggests a maternal defect in chromosome segregation fidelity.

### 2.2 Chromosomal karyotype analysis of proband peripheral blood

**Figure 2:**
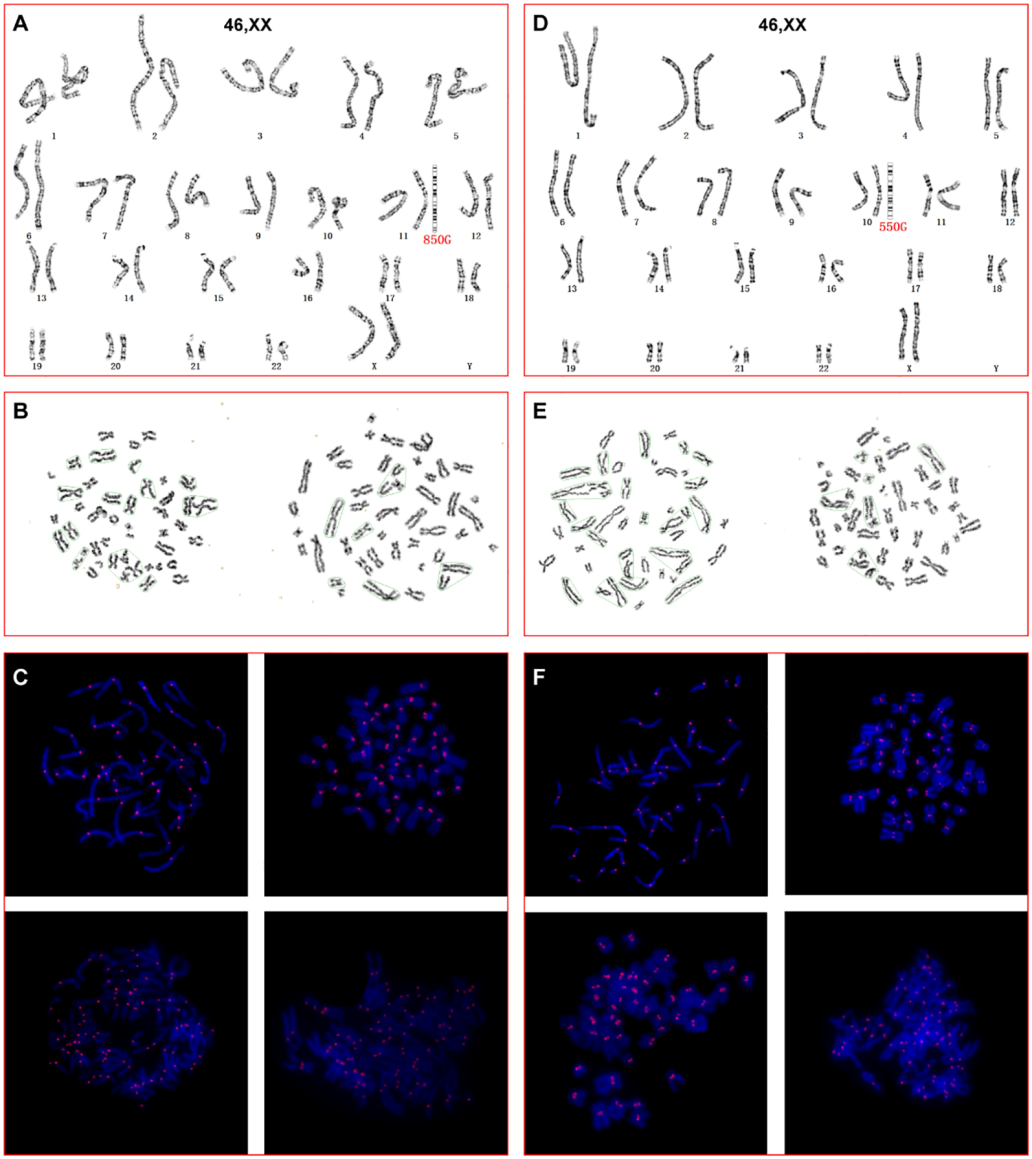
Chromosomal karyotype and centromere-specific fluorescence *in situ* hybridization (PNA-FISH) analyses of the two probands. **(A)** G-banded karyotype of peripheral blood lymphocytes from the proband in Family 1, showing no apparent chromosomal abnormalities. **(B)** Karyotype from the same proband revealing multiple chromosomes with prematurely separated sister chromatids—characteristic of the PCS phenotype. **(C)** Centromere-specific PNA-FISH analysis of the proband in Family 1, using a Cy3-labeled centromeric PNA probe (red signals) with DAPI counterstaining of nuclei (blue). Two distinct, well-separated centromeric signals are visible on multiple chromosomes, confirming PCS. **(D)** G-banded karyotype of peripheral blood lymphocytes from the proband in Family 2, also showing no obvious chromosomal abnormalities. **(E)** Karyotype from this proband demonstrating multiple chromosomes exhibiting premature centromere separation and split sister chromatids, consistent with PCS. **(F)** PNA-FISH result for the Family 2 proband, using the same Cy3-labeled centromeric probe (red) and DAPI (blue). Clearly separated paired centromere signals on several chromosomes further validate the presence of PCS.

To investigate the underlying cause of recurrent embryonic aneuploidy in the two probands, we performed high-resolution G-banded karyotyping on peripheral blood lymphocytes from both individuals. No overt numerical or structural chromosomal abnormalities were detected in either proband (**Figures 2A, D**). However, premature chromatid separation (PCS) was observed in a notable proportion of metaphase spreads: 8.93% (5 out of 56 cells) in Proband 1 and 13.79% (8 out of 58 cells) in Proband 2. These frequencies significantly exceed the control mean (0.40 ± 1.05%). In comparison, the mean G-band PCS rate among 20 healthy controls (10 male, 10 female; 50 cells scored per subject) was 0.40 ± 1.05% (range 0–4%). Single-sample t-tests confirmed that the PCS rates of both probands were significantly elevated relative to the control mean: Proband 1, 8.93% vs. 0.40 ± 1.05% (P < 0.001); Proband 2, 13.79% vs. 0.40 ± 1.05% (P < 0.001). Representative karyotypes showing PCS are presented in **Figures 2B, E**.

### 2.3 PNA-FISH confirmation of premature sister chromatid separation

To further validate the karyotype findings and obtain a more precise quantification of PCS frequency, we performed centromere-specific peptide nucleic acid fluorescence *in situ* hybridization (PNA-FISH) on metaphase spreads from both probands. The results clearly demonstrated that, in cells from each proband, numerous chromosomes (≥10 per cell) exhibited two spatially distinct centromeric signals, indicative of premature separation of sister chromatids (**Figures 2C, F**). This observation was fully consistent with the PCS phenotype identified by conventional karyotyping. In each case, ≥100 metaphase cells were scored: Proband 1 exhibited a FISH PCS rate of 18.58% (21/113 cells), and Proband 2 exhibited a FISH PCS rate of 24.43% (32/131 cells). The mean FISH PCS rate among the 20 healthy controls was 2.95 ± 0.60% (range 2–4%; 100 cells scored per subject). Single-sample t-tests confirmed that both probands were significantly elevated relative to the control mean: Proband 1, 18.58% vs. 2.95 ± 0.60% (P < 0.001); Proband 2, 24.43% vs. 2.95 ± 0.60% (P < 0.001). Detailed results from both karyotype and FISH analyses are summarized in **Table 2**.

**Table 2:** Results of chromosomal karyotype and FISH analyses in the probands.

Family	CV/SV	PCS (Karyotyping)	PCS (FISH)
Family 1	None	8.93% (5/56)	18.58% (21/113)
Family 2	None	13.79% (8/58)	24.43% (32/131)

### 2.4 Whole-exome sequencing and Sanger validation

**Figure 3:**
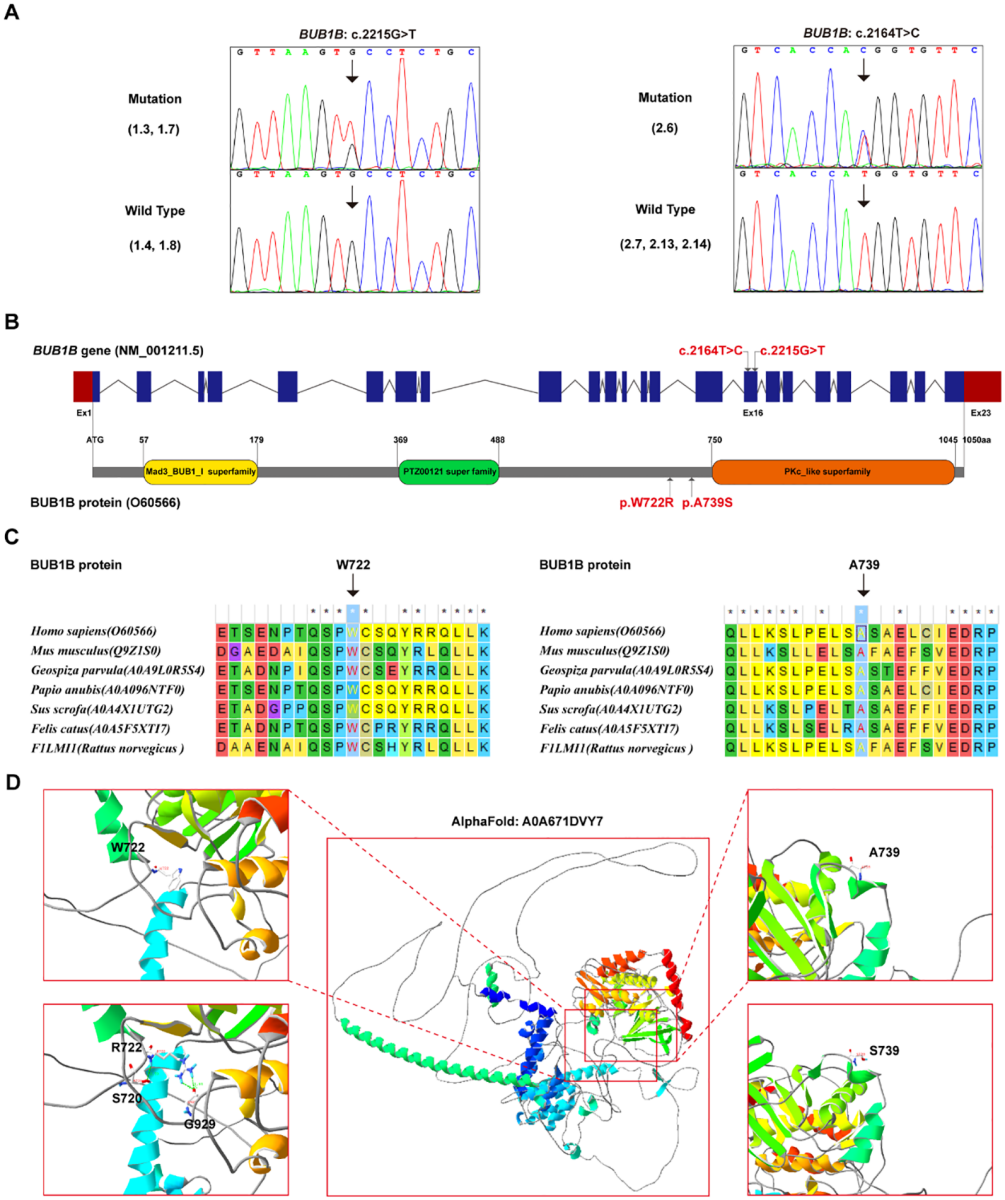
Validation and functional prediction of *BUB1B* variants. **(A)** Sanger sequencing chromatograms confirming the *BUB1B* variants in the probands and available family members. Arrows indicate the variant positions. The left panel shows the c.2215G>T (p.A739S) variant in Family 1; the right panel shows the c.2164T>C (p.W722R) variant in Family 2. Sequencing traces confirm variant authenticity and co-segregation with the RPL phenotype. **(B)** Schematic representation of the *BUB1B* gene and BUBR1 protein domain architecture, with the locations of the two identified missense variants (p.W722R and p.A739S) indicated. Both residues map adjacent to the kinase domain of BUBR1. **(C)** Multiple sequence alignment showing evolutionary conservation of the affected amino acid residues across species (from zebrafish to human). Both tryptophan-722 and alanine-739 are highly conserved throughout evolution. **(D)** Predicted three-dimensional structural models of the BUBR1 kinase domain. Top left: wild-type; bottom left: p.W722R mutant. Substitution of the large hydrophobic aromatic tryptophan with a positively charged, hydrophilic arginine may disrupt local packing and introduce new hydrogen bonds (dashed lines), potentially compromising protein stability or activity. Top right: wild-type; bottom right: p.A739S mutant. Replacement of the small methyl side chain of alanine with the hydroxymethyl group of serine could create new hydrogen-bonding opportunities or alter local surface electrostatics.

Whole-exome sequencing (WES) was performed on both probands, with a focused analysis on genes involved in chromosome segregation and spindle assembly checkpoint (SAC) function. In the proband from Family 1, we identified a novel heterozygous missense variant in *BUB1B*: c.2215G>T (p.A739S). This variant is extremely rare in the gnomAD database, absent from ClinVar, and consistently predicted to be deleterious by multiple in silico tools. According to the ACMG/AMP guidelines, this variant was classified as a variant of uncertain significance (VUS), as only PM2 (absent/rare in population databases) and PP3 (multiple in silico predictions of pathogenicity) criteria were met, which is insufficient for a likely pathogenic classification. Sanger sequencing confirmed the presence of the variant, and segregation analysis revealed that it was inherited from the proband’s father; neither the mother nor the younger brother carried the variant (**Figure 3A**, left panel).

In the proband from Family 2, we identified another novel heterozygous missense variant in *BUB1B*: c.2164T>C (p.W722R). This variant is also extremely rare in gnomAD, not reported in ClinVar, and similarly predicted to be damaging by SIFT, PolyPhen-2, and MutationTaster2. Applying the ACMG/AMP framework, this variant was classified as a variant of uncertain significance (VUS), as only PM2 (absent/rare in population databases) and PP3 (multiple in silico predictions of pathogenicity) criteria were met, which is insufficient for a likely pathogenic classification. Sanger sequencing validated the variant, and family screening showed that the proband’s sister—who has no history of pregnancy loss—and her two sons did not carry the variant (**Figure 3A**, right panel).

### 2.5 Evolutionary conservation and structural impact of the missense variants

We performed in-depth bioinformatic analyses on the two identified *BUB1B* missense variants: c.2164T>C (p.W722R) and c.2215G>T (p.A739S). Both variants reside in exon 16 of *BUB1B* (**Figure 3B**) and affect residues located within or adjacent to the protein’s kinase domain. Multiple sequence alignment across diverse species demonstrated that both tryptophan-722 and alanine-739 are highly evolutionarily conserved, underscoring their functional importance (**Figure 3C**).

Furthermore, homology-based three-dimensional structural modeling revealed significant biophysical alterations caused by these substitutions (**Figure 3D**). The p.W722R mutation replaces a large, hydrophobic, aromatic tryptophan residue with a positively charged, hydrophilic arginine. This substitution not only introduces substantial steric bulk but—more critically—disrupts the integrity of a local hydrophobic core essential for structural stability. The newly introduced arginine side chain forms ectopic hydrogen bonds with neighboring residues, creating an unnatural polar microenvironment. This forced polarity likely induces local conformational strain or distortion, thereby compromising the structural stability of the kinase domain and potentially impairing BUBR1’s catalytic activity or regulatory function.

In contrast, the p.A739S substitution exerts a more localized and subtle effect. The replacement of alanine’s small, nonpolar methyl side chain with serine’s hydroxymethyl group converts the residue from a purely hydrophobic character to one capable of polarity and hydrogen bonding (**Figure 3D**, right). Although this change is unlikely to cause major global structural rearrangements, it significantly increases the polarity of a surface-exposed loop region. This altered physicochemical property may specifically interfere with BUBR1’s interaction with particular binding partners or downstream effector molecules—either by forming new hydrogen bonds or by modifying the local electrostatic surface potential—thereby potentially disrupting signaling fidelity within the SAC pathway.

### 2.6 RNA expression and protein level analysis of *BUB1B* variants

**Figure 4:**
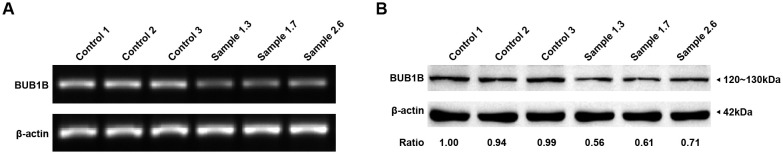
RNA expression and protein level analysis of *BUB1B* variants in peripheral blood mononuclear cells. **(A)** Agarose gel electrophoresis of RT-PCR products resolved on a 2% agarose gel; β-actin amplicons served as a loading reference. Band intensity in both probands appeared visually reduced relative to the control lanes; the paternal carrier showed a similar trend toward reduced expression. **(B)** Western blot analysis of BUBR1 protein expression. Whole-cell lysates were probed with an anti-BUBR1 antibody; β-actin was used as a loading control. Both probands exhibited a trend toward reduced BUBR1 band intensity compared to controls; the paternal carrier showed a similar trend toward reduced BUBR1 band intensity.

To investigate whether the identified *BUB1B* variants affect gene expression at the RNA and protein levels, we performed agarose gel electrophoresis and Western blot analyses on peripheral blood mononuclear cells (PBMCs) from both probands, the clinically unaffected father of Proband 1 (a heterozygous carrier), and three healthy controls. Agarose gel electrophoresis of RT-PCR products showed visually diminished *BUB1B*-specific band intensity in both probands relative to the control lanes, while β-actin bands remained comparable across all samples (**Figure 4A**). The father of Proband 1 also showed a similar trend toward reduced *BUB1B* band intensity relative to controls. At the protein level, Western blot analysis demonstrated a trend toward decreased BUBR1 protein abundance in both probands, with β-actin serving as a loading control (**Figure 4B**). The father of Proband 1 also exhibited a similar trend toward reduced BUBR1 band intensity relative to controls. Given the qualitative nature of these assays and the inherent variability of lymphocyte-based expression analyses, these findings should be interpreted as preliminary evidence of reduced *BUB1B/*BUBR1 expression rather than definitive quantification. Nevertheless, the concordant reduction observed at both the mRNA and protein levels across two independent probands carrying distinct *BUB1B* variants is consistent with a dose-sensitive haploinsufficiency model and supports the hypothesis that heterozygous missense variants in *BUB1B* reduce BUBR1 below a functional threshold sufficient to compromise SAC fidelity in the female germline.

## 3 Discussion

*BUB1B* (encoding BUBR1) is a core component of the spindle assembly checkpoint (SAC), a surveillance mechanism that ensures accurate chromosome segregation during mitosis. Its primary role is to monitor proper kinetochore–microtubule attachments and prevent premature separation of sister chromatids until bipolar attachment is achieved. BUBR1 enforces this checkpoint by inhibiting the anaphase-promoting complex/cyclosome (APC/C), thereby delaying anaphase onset until all chromosomes are correctly aligned. More recently, BUBR1 has also been shown to negatively regulate centrosome duplication during interphase by suppressing Polo-like kinase 1 (Plk1) activity, thus preventing centrosome amplification and maintaining genomic stability (18). When *BUB1B* function is compromised, SAC fidelity is weakened, leading to increased chromosome missegregation, centrosome abnormalities, and aneuploidy—collectively forming a critical genetic basis for embryonic developmental failure and pregnancy loss.

Historically, research on *BUB1B* has focused on its roles in cancer and mosaic variegated aneuploidy (MVA) syndrome. MVA is a rare autosomal recessive disorder characterized by widespread aneuploidy, growth retardation, microcephaly, and predisposition to early-onset malignancies, typically caused by biallelic pathogenic *BUB1B* mutations (3, 10, 11). However, emerging evidence suggests that heterozygous *BUB1B* variants may act in a dominant manner to contribute to various reproductive disorders. For instance, missense *BUB1B* variants have been linked to premature ovarian insufficiency (POI), with carriers exhibiting menstrual irregularities, elevated FSH levels, and diminished ovarian reserve (12). Additionally, *BUB1B* mutations have been reported at an elevated frequency in patients with unexplained recurrent spontaneous abortion (URSA), implicating the gene in embryonic aneuploidogenesis (4). Nevertheless, a definitive causal relationship between heterozygous *BUB1B* variants and RPL remains inadequately established, particularly due to the lack of familial co-segregation data and functional validation.

In this study, we performed comprehensive analyses of two Han Chinese families affected by RPL. We observed significantly elevated rates of premature chromatid separation (PCS) in peripheral blood lymphocytes of both probands and confirmed, via chromosomal microarray analysis (CMA), that all six miscarriage specimens exhibited random aneuploidies—strongly indicating a maternal defect in chromosome segregation. Whole-exome sequencing (WES) identified two novel heterozygous missense variants in exon 16 of *BUB1B*: c.2164T>C (p.W722R) and c.2215G>T (p.A739S). Both residues lie within the highly conserved kinase domain and were consistently predicted as deleterious by multiple bioinformatic tools. Structural modeling suggested that these substitutions likely disrupt protein conformation—either by perturbing a hydrophobic core (p.W722R) or by introducing aberrant hydrogen-bonding potential (p.A739S)—thereby impairing BUBR1 stability and function.

Several prior reports have documented *BUB1B* variants in the context of pregnancy loss and reproductive failure. Schmid et al. reported pregnancy loss in human carriers associated with BUBR1 deficiency in mouse models (11), and Matsuura et al. described seven Japanese families with monoallelic *BUB1B* mutations exhibiting PCS syndrome, with PCS traits observed in carrier parents (3). More recently, Berkay et al. identified *BUB1B* among candidate genes in a whole-exome sequencing study of unexplained RPL and implantation failure, further implicating spindle assembly checkpoint dysfunction in reproductive failure (19). Mou et al. also reported *BUB1B* genetic polymorphisms in a cohort of unexplained recurrent spontaneous abortion (4). Taken together, these reports support a growing body of evidence linking *BUB1B* dysfunction to reproductive failure, and our study provides the first direct familial evidence connecting heterozygous *BUB1B* missense variants with RPL in Han Chinese families.

Notably, Bohers et al. previously demonstrated in cell models that graded reduction of BUBR1 protein levels proportionally increases PCS frequency and aneuploidy (13). Our clinical findings provide direct human evidence supporting this dose-sensitive model: even heterozygous missense variants may reduce BUBR1 below a critical threshold, compromising SAC stringency (8). This allows chromosomes with unattached or improperly attached kinetochores to prematurely enter anaphase, resulting in aneuploid gametes and embryos. Critically, segregation analysis revealed that in Family 1, the variant was inherited from the clinically unaffected father, suggesting sex-specific or incomplete penetrance; in Family 2, unaffected relatives (including the proband’s sister and her two sons) did not carry the variant—consistent with the hypothesis that the variant contributes to RPL specifically in the female germline context. Together, these data offer suggestive genetic evidence that heterozygous *BUB1B* variants can underlie RPL and expand the known phenotypic spectrum of *BUB1B*-related disorders. Importantly, our agarose gel electrophoresis and Western blot analyses revealed a concordant trend toward reduced *BUB1B* mRNA and BUBR1 protein levels in both probands’ peripheral blood lymphocytes, providing preliminary functional evidence that these missense variants impair *BUB1B* expression and are consistent with a haploinsufficiency mechanism. Although these assays are qualitative in nature, the convergent reduction at both the transcript and protein levels across two independent families strengthens the biological plausibility of the dose-sensitive model proposed by Bohers et al (13).

The observation that the c.2215G>T(p.A739S) variant in Family 1 was inherited from the clinically unaffected father raises an important question regarding sex-specific penetrance. Several lines of evidence suggest that this reflects the uniquely high demand for BUBR1 protein in female meiosis. In mouse oocytes, BUBR1 protein is essential for sustained spindle assembly checkpoint (SAC) activity, accurate timing of meiosis I, and the establishment of robust kinetochore–microtubule attachments (20, 21). Critically, BUBR1 protein levels increase progressively during oocyte meiotic maturation from the germinal vesicle to metaphase II stage (22), indicating that oocytes impose a higher quantitative demand on BUBR1 than somatic or male germ cells. The SAC in mammalian oocytes is inherently less stringent than in mitotic cells and cannot respond to a single unattached kinetochore (20); consequently, even a partial reduction in BUBR1 abundance—as expected from a heterozygous missense variant—may be sufficient to compromise the already-fragile oocyte SAC, leading to premature anaphase onset, PCS, and aneuploid embryos. In contrast, male germ cells do not share this dependency: spermatocytes tolerate reduced SAC protein dosage without detectable chromosomal defects, consistent with the reproductive normality of the father in Family 1.

Based on our findings, we recommend that women with RPL who carry *BUB1B* VUS or pathogenic variants undergo preimplantation genetic testing for aneuploidy (PGT-A) to select euploid embryos for transfer, thereby improving live birth rates and reducing miscarriage risk. Importantly, given the established link between *BUB1B* variants and POI (12), if patients repeatedly fail to yield transferable euploid embryos or exhibit markedly diminished ovarian reserve, donor egg IVF should be considered.

This study has several limitations. First, although PCS frequencies in peripheral blood lymphocytes were significantly above the normal threshold, these somatic cells may not fully recapitulate chromosome segregation dynamics in oocytes. While centromere-specific PNA-FISH enhanced detection accuracy, the method still relies on mitotic metaphase spreads and may introduce selection bias. Given the universal aneuploidy observed in miscarriage tissues, it is plausible that BUBR1 dysfunction is more pronounced in the germline. Second, our cohort is limited to two families; larger population-based studies are needed to determine the prevalence and pathogenic contribution of *BUB1B* variants in RPL. Third, while we performed agarose gel electrophoresis and Western blot analyses to assess *BUB1B* expression at the mRNA and protein levels—providing preliminary evidence of reduced expression in both probands—these assays are qualitative and were conducted in peripheral blood lymphocytes, which may not fully reflect expression dynamics in oocytes or early embryos. More rigorous functional studies, including engineered mutant cell lines, SAC activity measurements, and direct assessment of chromosomal stability, are still needed to establish the mechanistic link between these variants and SAC dysfunction. Future work could leverage organoid models, single-cell multi-omics, and CRISPR-based genome editing to dissect how specific *BUB1B* missense variants disrupt human oocyte maturation and early embryogenesis.

Finally, therapeutic strategies aimed at modulating SAC activity or optimizing PGT-A protocols in assisted reproduction may offer new hope for couples carrying such variants. Most importantly, our results indicate that *BUB1B* should be considered in targeted genetic screening for RPL patients presenting with unexplained recurrent loss and a cytogenetic PCS phenotype, rather than as a routine screen for all RPL cases (5, 23). With decreasing costs of whole-exome sequencing and wider adoption of ACMG variant interpretation guidelines (14), such precision diagnostics are becoming increasingly feasible—and hold promise for significantly improving reproductive outcomes in affected individuals. It should be noted that the paternal inheritance of the variant in Family 1 highlights that *BUB1B* variants may be transmitted from clinically unaffected male carriers, which has important implications for variant interpretation and family counseling.

## 4 Conclusions

This study is the first to establish, at the familial level, a potential causal link between heterozygous missense variants in *BUB1B* (c.2164T>C and c.2215G>T) and unexplained recurrent pregnancy loss (RPL). The significantly elevated PCS rates in the probands’ peripheral blood, the widespread random aneuploidies observed in all miscarriage specimens, and the segregation analysis showing variant absence in unaffected relatives collectively support the hypothesis that *BUB1B* dysfunction leads to a maternal chromosome segregation defect. Bioinformatic analyses further indicate that both variants affect highly evolutionarily conserved residues within the kinase domain and are likely to impair BUBR1’s SAC regulatory function by destabilizing its protein conformation, though their pathogenicity requires further functional validation given their current VUS classification. Preliminary functional analyses—including agarose gel electrophoresis and Western blot—revealed a concordant trend toward reduced *BUB1B* mRNA and BUBR1 protein expression in both probands, providing initial molecular support for a haploinsufficiency mechanism. These findings not only broaden the clinical phenotypic spectrum of *BUB1B*-related disorders but also provide a critical molecular basis for precision genetic counseling and personalized assisted reproductive strategies—such as preimplantation genetic testing for aneuploidy (PGT-A) or oocyte donation—for affected RPL patients. Future studies with larger cohorts and functional validation experiments are warranted to further elucidate the functional impact and pathogenic mechanisms of these variants.

## 5 Materials and methods

### 5.1 Study subjects

This study enrolled two unrelated Han Chinese families with recurrent pregnancy loss (RPL) (Family 1 and Family 2). Each family included a female proband who had experienced ≥3 clinically confirmed pregnancy losses, along with her spouse, parents, and siblings. All participants provided written informed consent, and the studies involving human participants were reviewed and approved by The Ethics Committee of Beijing Jiaen Hospital (Approval no. BJH-2025024). The definition of RPL followed the European Society of Human Reproduction and Embryology (ESHRE) guidelines (5). All study performed associated with human participants met ethical standards of the 2013 Declaration of Helsinki.

### 5.2 Sample collection and processing

Peripheral venous blood samples were collected from probands and family members using EDTA tubes for next-generation sequencing (NGS) and Sanger validation, and heparinized tubes for karyotyping and fluorescence *in situ* hybridization (FISH). Spontaneously aborted fetal tissues (gestational age 8–16 weeks) were immediately placed in RNAlater stabilization solution for subsequent chromosomal microarray analysis.

### 5.3 Chromosomal microarray analysis of products of conception

Genomic DNA was extracted from miscarriage tissues using the QIAGEN QIAamp Fast DNA Tissue Kit. DNA concentration was measured using a Thermo Fisher NanoDrop 2000 spectrophotometer and adjusted to 50 ng/μL. Genome-wide copy number variation (CNV) analysis, along with assessment of loss of heterozygosity (LOH) and uniparental disomy, was performed on six DNA samples using the Affymetrix CytoScan™ 750K Array. Library preparation—including restriction digestion, ligation, amplification, purification, fragmentation, and labeling—was carried out according to the manufacturer’s standard protocol using Affymetrix reagents and instrumentation. Labeled DNA samples were hybridized onto CytoScan 750K arrays at 50 °C with shaking at 60 rpm for 16–18 hours. After hybridization, chips were washed, stained, and scanned. Data were analyzed using Chromosome Analysis Suite (ChAS) v4.0 with GRCh37 as the reference genome and a resolution threshold of 200 kb (24, 25).

### 5.4 High-resolution chromosome karyotyping

Heparinized whole blood (0.45 mL) was inoculated into lymphocyte culture medium under a biosafety cabinet following the American Society of Clinical Laboratory Science (ASCLS) cytogenetics laboratory manual. Cultures were incubated at 37 °C for 72 hours. Metaphase cells were harvested, processed through standard colchicine treatment, hypotonic lysis, fixation, G-banding, and slide preparation. Slides were scanned using a Leica GSL-120 automated cytogenetic imaging system, and karyotypes were analyzed with CytoVision software, followed by manual verification by certified cytogeneticists. Chromosomal abnormalities were assessed, and the proportion of metaphase spreads exhibiting premature chromatid separation (PCS) was quantified. Twenty healthy volunteers (10 male, 10 female; age range 25–38 years) with no personal or family history of recurrent pregnancy loss, chromosomal abnormalities, or known genetic disorders were enrolled as controls and subjected to the same karyotyping procedure. For each control subject, 50 metaphase cells were scored for PCS frequency to establish the reference distribution.

### 5.5 Centromere-specific peptide nucleic acid fluorescence *in situ* hybridization

FISH was performed using a Cy3-labeled PNA probe targeting human α-satellite DNA (PNA Bio Inc.) to enhance centromere resolution (4). Fixed interphase or metaphase cells on slides were dehydrated through an ethanol series (70%, 90%, and 100%). Immediately before hybridization, slides were heat-denatured at 82 °C for 3–5 minutes. A 10 μL hybridization mix containing 2 μL of CENT-Cy3 PNA probe (final concentration 50–200 nM) and 8 μL of hybridization buffer (70% formamide, 10 mM Tris–HCl pH 7.5, 1% bovine serum albumin, and 0.5% blocking reagent) was applied per slide. Hybridization was carried out in a humidified chamber at 37 °C in the dark for 30–90 minutes. Slides were then washed stringently, counterstained with DAPI-containing antifade mounting medium, and coverslipped. For each sample, ≥100 metaphase cells were scored. PCS was defined as the presence of ≥10 chromosomes per cell showing clearly separated sister chromatids with two distinct centromeric signals (2). The same 20 healthy control subjects underwent identical PNA-FISH analysis; 100 metaphase cells were scored per subject. PCS frequencies from controls were used to establish the reference distribution for statistical comparison with the probands. Statistical significance was assessed using a one-sample t-test (two-tailed), with the proband’s PCS rate compared against the control mean ± SD (significance threshold: P < 0.05).

### 5.6 Whole-exome sequencing and bioinformatic analysis

WES was performed on an Illumina NovaSeq 6000 platform using the Agilent SureSelect XT Human All Exon Kit for target enrichment, with paired-end 150-bp (PE150) sequencing. Raw reads (with >89% bases at Q30 quality) were aligned to the human reference genome (hg19/GRCh37) using the Burrows-Wheeler Aligner (BWA) (26). PCR duplicates were removed using Picard v1.57. Variants were annotated using public databases including ClinVar and gnomAD, and classified according to ACMG/AMP guidelines (14). Pathogenicity of missense variants was predicted using SIFT (15), PolyPhen-2 (16), and MutationTaster2 (17). Priority was given to genes involved in chromosome segregation, meiosis, and the spindle assembly checkpoint (SAC) pathway (e.g., *BUB1B*, *MAD2L1, TTK*) (9). Candidate variants were validated by Sanger sequencing (ABI 3730xl) and tested for co-segregation within families.

### 5.7 Conservation and structural analysis of missense variants

Evolutionary conservation of the affected amino acid residues was assessed using MEGA7(https://www.megasoftware.net/) with default parameters. Three-dimensional structural models of wild-type (WT) and mutant (MT) BUBR1 proteins harboring the identified missense variants were generated and compared using the SWISS-MODEL web server (https://swissmodel.expasy.org/) with default settings.

### 5.8 RNA extraction and agarose gel electrophoresis

Total RNA was extracted from peripheral blood mononuclear cells (PBMCs) of both probands, the father of Proband 1, and three healthy controls using TRIzol reagent (Thermo Fisher Scientific) according to the manufacturer’s protocol. RNA concentration and purity were assessed using a NanoDrop 2000 spectrophotometer (A260/A280 ratio ≥ 1.8). First-strand cDNA was synthesized from 1 μg of total RNA using the PrimeScript RT Reagent Kit with gDNA Eraser (Takara Bio). RT-PCR was performed using primers targeting *BUB1B* (forward: 5′-GCAGCAGAAGAAGCAGGTCA-3′; reverse: 5′-TGGTCTTCAGCTTCTGCACC-3′) and the reference gene β-actin (forward: 5′-CATGTACGTTGCTATCCAGGC-3′; reverse: 5′-CTCCTTAATGTCACGCACGAT-3′). RT-PCR products were resolved on a 2% agarose gel stained with ethidium bromide and visualized under UV illumination, with β-actin amplicons serving as a loading reference. Given the small sample size, results are interpreted qualitatively as indicative of expression trends.

### 5.9 Western blot analysis

Whole-cell protein lysates were prepared from PBMCs of both probands, the father of Proband 1, and three healthy controls using RIPA lysis buffer supplemented with protease and phosphatase inhibitor cocktail (Roche). Protein concentration was determined by the BCA assay (Thermo Fisher Scientific). Equal amounts of protein (30 μg per lane) were separated by 8% SDS-PAGE and transferred onto PVDF membranes (Millipore). Membranes were blocked with 5% non-fat dry milk in TBST for 1 hour at room temperature, then incubated overnight at 4 °C with primary antibodies: anti-BUBR1 (rabbit monoclonal, Abcam ab254326, 1:1000) and anti-β-actin (mouse monoclonal, Sigma-Aldrich A5441, 1:5000). After washing, membranes were incubated with HRP-conjugated secondary antibodies for 1 hour at room temperature. Bands were detected using enhanced chemiluminescence (ECL) reagent (Thermo Fisher Scientific) and imaged on a ChemiDoc MP Imaging System (Bio-Rad). Band intensity was assessed visually; β-actin served as a loading control. Due to the qualitative nature of the assay and the limited sample size, results are reported as indicative trends rather than quantitative measurements.

## Data Availability

The original contributions presented in the study are included in the article/supplementary material. Further inquiries can be directed to the corresponding authors.
